# Supplementary figures and images for: Quantifying morphological variation in the Castilleja pilosa species complex (Orobanchaceae)
Source: PeerJ. 2019 Jun 20;7:e7090. doi: 10.7717/peerj.7090 (PMC6589334; doi:10.7717/peerj.7090)

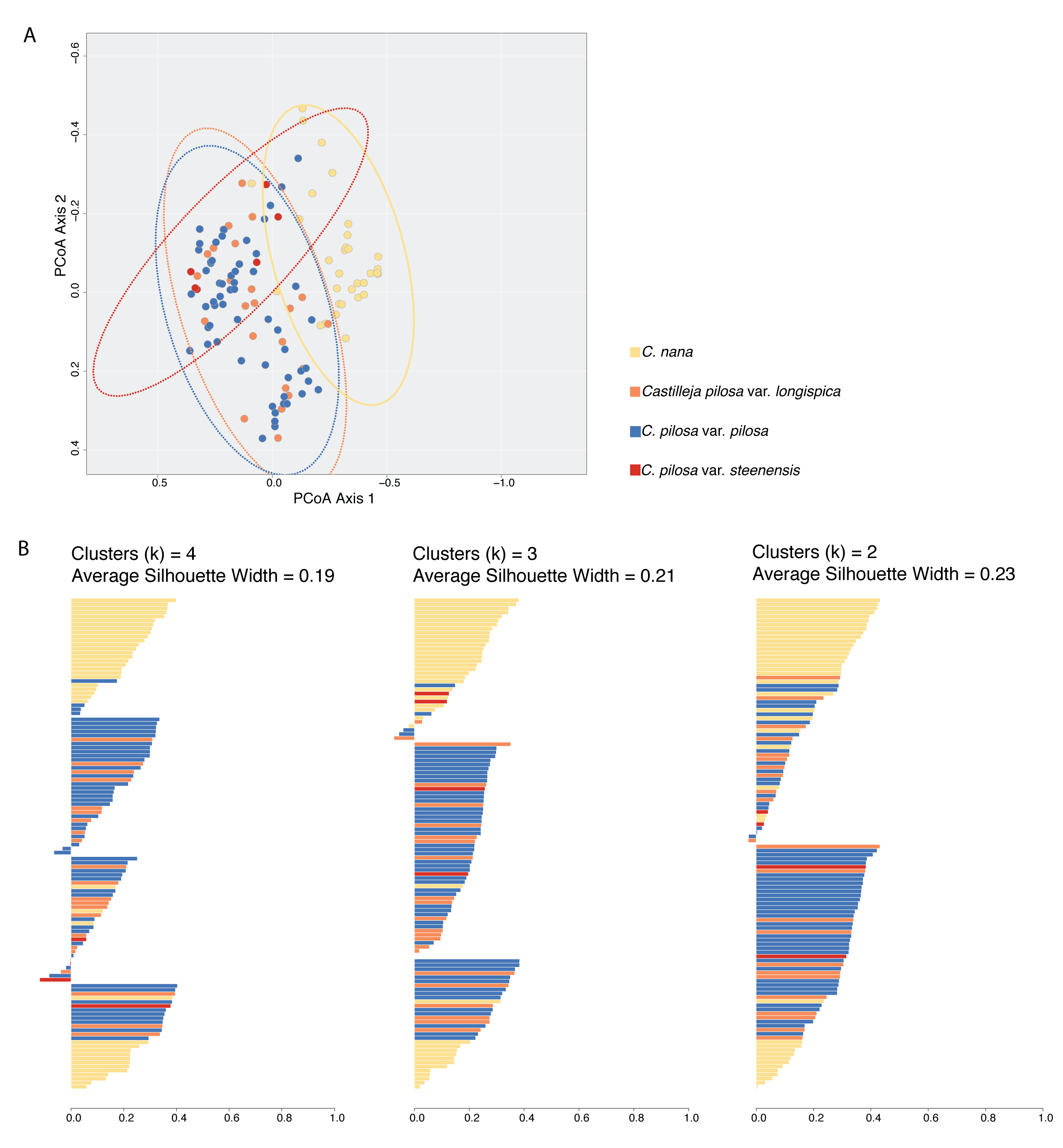

Supplement: Supplemental Information 1 — Results of Principal Coordinates Analysis (PCoA) (A) and fuzzy-clustering analyses (B) where no outliers were removed from theanalysis. The PCoA plot shows the first two axes of variation where individuals are plotted in morphospace, and colored according to species identification. Results of fuzzy clustering for k = 4 clusters (left), k = 3 clusters (middle), and k = 2 clusters (right). For each set of silhouettes, the width of each bar corresponds to the silhouette coefficient for that individual in the analysis; average silhouette coefficient for each analysis (k = 4, 3, 2) is reported. Bars are painted with colors corresponding to species identification. In both plots, yellow = C. nana, blue = C. pilosa var. pilosa, orange = C. pilosa var. longispica, red = C. pilosa var. steenensis. [file peerj-07-7090-s001.png]

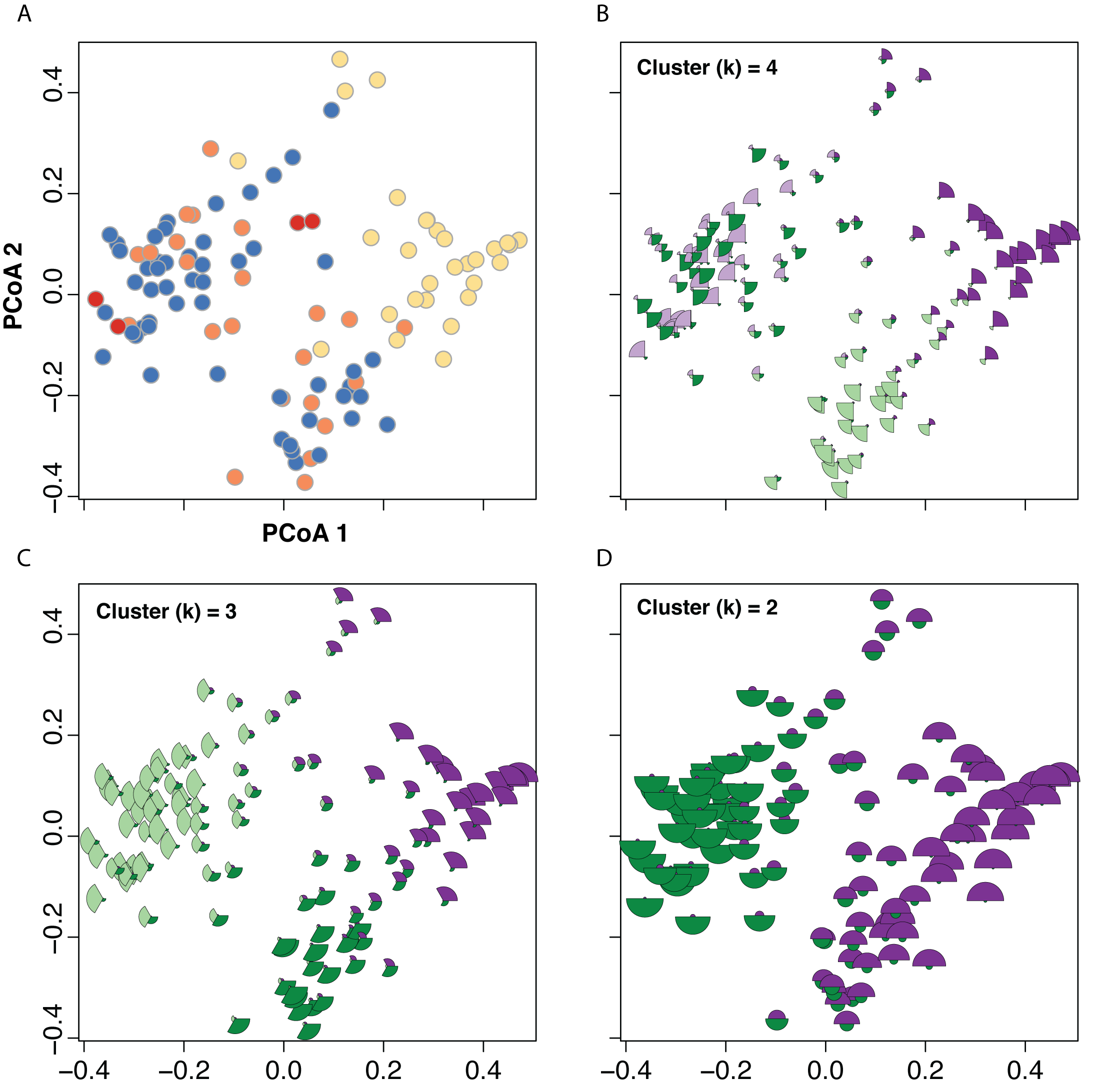

Supplement: Supplemental Information 2 — Results of Principal Coordinates Analysis (PCoA; A) and the mapping of assignment probabilities from fuzzy clustering analyses onto points in morphospace (B–D). In each plot, each point is divided into segments corresponding to recovered clusters (identified by different colors). The size of the segment corresponds to the assignment probability of that individual to that cluster—larger segments correspond to higher assignment probabilities and smaller segments correspond to lower assignment probabilities. The purple and green colors only serve to distinguish different clusters; the top left panel (with individuals painted as yellow = C. nana, blue = C. pilosa var. pilosa, orange = C. pilosa var. longispica, and red = C. steenensis) serves as a reference for the taxonomic identification of each individuals. [file peerj-07-7090-s002.png]

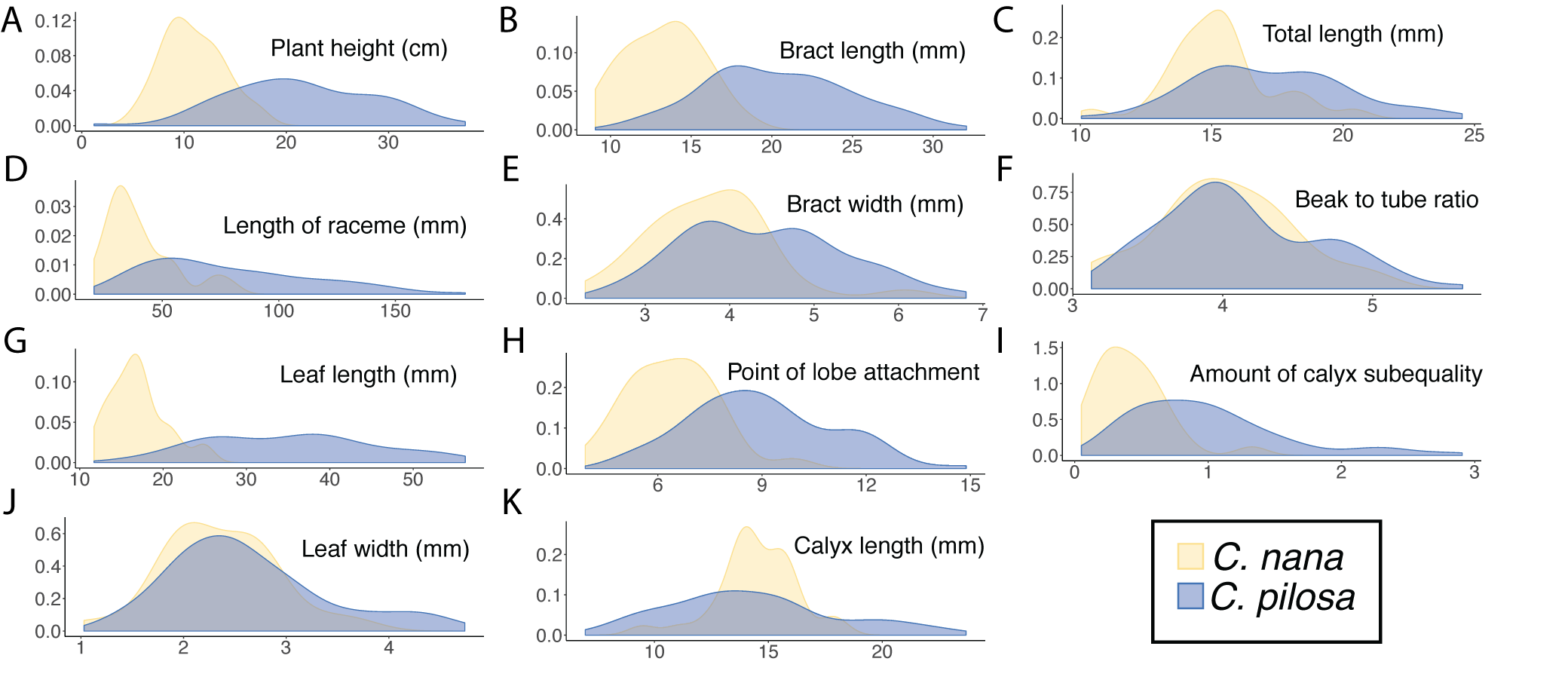

Supplement: Supplemental Information 3 — Kernel density estimates of raw trait values for the continuous traits measured in this study (A-K), organized by species. C. pilosa (including C. pilosa var. longispica, C. pilosa var. pilosa, and C. pilosa var. steenensis) (blue) and C. nana (yellow). [file peerj-07-7090-s003.png]
